# Brain Entropy During Aging Through a Free Energy Principle Approach

**DOI:** 10.3389/fnhum.2021.647513

**Published:** 2021-03-22

**Authors:** Filippo Cieri, Xiaowei Zhuang, Jessica Z. K. Caldwell, Dietmar Cordes

**Affiliations:** Department of Neurology, Cleveland Clinic Lou Ruvo Center for Brain Health, Las Vegas, NV, United States

**Keywords:** brain entropy, entropic brain, aging, fMRI, free energy, Alzheimer, neuroaging

## Abstract

Neural complexity and brain entropy (BEN) have gained greater interest in recent years. The dynamics of neural signals and their relations with information processing continue to be investigated through different measures in a variety of noteworthy studies. The BEN of spontaneous neural activity decreases during states of reduced consciousness. This evidence has been showed in primary consciousness states, such as psychedelic states, under the name of “the entropic brain hypothesis.” In this manuscript we propose an extension of this hypothesis to physiological and pathological aging. We review this particular facet of the complexity of the brain, mentioning studies that have investigated BEN in primary consciousness states, and extending this view to the field of neuroaging with a focus on resting-state functional Magnetic Resonance Imaging. We first introduce historic and conceptual ideas about entropy and neural complexity, treating the mindbrain as a complex nonlinear dynamic adaptive system, in light of the free energy principle. Then, we review the studies in this field, analyzing the idea that the aim of the neurocognitive system is to maintain a dynamic state of balance between order and chaos, both in terms of dynamics of neural signals and functional connectivity. In our exploration we will review studies both on acute psychedelic states and more chronic psychotic states and traits, such as those in schizophrenia, in order to show the increase of entropy in those states. Then we extend our exploration to physiological and pathological aging, where BEN is reduced. Finally, we propose an interpretation of these results, defining a general trend of BEN in primary states and cognitive aging.

The pessimist complains about the wind, the optimist waits for the wind to change; the realist adapts the sails (Chamfort, 1795/1969).

## Introduction

Several factors characterize the human brain as one of the most complex organs in nature. The first is the billions of neurons from which it is composed, and which are associated with high total energy costs, expressed in the brain’s use of 20% of the body’s total energy, despite representing only 2% of a person’s total body weight (Raichle and Gusnard, 2002). The number of neurons in an individual’s brain is generally (and perhaps simplistically) assumed to be a determinant factor for the computational power and expressive capability of the neurocognitive system. These billions of neurons are frequently and evocatively compared to the number of stars in the Milky Way, which contains an estimated 200–400 billion stars of different brightness and size. This number is not so far from the number of cells in the human brain. Our nervous system in fact has about 10^14^ synapses linking approximately 86.1 ± 8.1 billion neuronal and 84.6 ± 9.8 billion nonneuronal cells (Azevedo et al., 2009). Less than 20% of neurons are located in the cerebral cortex, with the ratios between glial cells and neurons similar to those found in other primates, calling into question the classical view that humans stand out from other primates in their structural brain composition, rather indicating that concerning numbers of neuronal and nonneuronal cells, the human brain is an isometrically scaled-up primate brain (Azevedo et al., 2009).

A second contributor to the complexity of the human brain is the fact that it expresses more of the total genetic information encoded in DNA than does any other organ in the human body (Kandel et al., 2000). Between 30 and 50% of ∼25,000 known protein coding genes are expressed in the human brain, the highest level of gene expression compared with other mammals and primates (Cáceres et al., 2003).

The last characteristic associated with the complexity of the neurocognitive system—the most relevant to this manuscript and certainly the most tautological—concerns its complex behavior, which recently led to the hypothesis of the *entropic brain* (Carhart-Harris et al., 2014; Carhart-Harris, 2018), where the term entropy refers to the second law of thermodynamics, formulated in 1851 by Clausius, after Carnot’s work. In this context, the quality of different neurocognitive states can be measurable by the entropic level, in a given parameter of spontaneous brain activity, through electroencephalogram (EEG), Magnetoencephalography (MEG), or the oxygen level-dependent (BOLD) resting state signal of the functional Magnetic Resonance Imaging (rs-fMRI). We focus our attention on this latest approach.

Entropy, disorder, uncertainty, and complexity often are used as synonymous in neuroscientific context and actually there is an unquestionable connection between informational uncertainty and physical disorder, with an underlying unity linking generative processes of adaptation, mind, and life (Friston et al., 2012a). Entropy is a measure of uncertainty and out of thermodynamics, John Neumann ironically suggested to Claude Shannon his new uncertainty measure’s name:

“*You should call it entropy, for two reasons: in the first place your uncertainty has been used in statistical mechanics under that name, so it already has a name. In the second place, and more important, no one really knows what entropy really is, so in a debate you will always have the advantage*” (cited in Tribus and McIrvine, 1971).

John Neumann’s words are true especially when this idea is applied to the brain entropy (BEN). In this paper we review the studies that have investigated this complexity. Through the review of these works, we also describe the neurocognitive^1^ system as engaged to reach a difficult balance between order and chaos.

As a measure of uncertainty, Pincus (1991) claimed that entropy measures the randomness and predictability of stochastic processes, generally increasing with greater randomness, where more entropy corresponds to greater complexity. In order to describe the BEN within the neurocognitive system, it is useful to define some characteristics of the system.

*Alive complex biological systems* have specific features, such as a boundary, in principle able to distinguish an inside and an outside of the system, allowing the differentiation between the organism and the “non-organism.” Friston (2010) propose the assumption to adopt the statistical tool of the Markov blanket to describe this characteristic^2^. The system is composed by simpler components (in the case of the brain, for example, neurons, synapses, networks, etc.), which can interact and communicate with one another in hierarchical and bidirectional ways (Kelso et al., 2009), with feedback mechanisms^3^. Also, the components are in nonlinear dynamic relationship, with non-proportional interaction between input and output^4^. Related to this feature, the system has a hierarchical and emergent behavior, in which the whole model can behave in new and different ways than the hierarchically underlying components. Moreover, the system has the characteristic of autopoiesis (Maturana and Varela, 1980), which refers to a system capable of reproducing and maintaining itself, allowing its evolution. The system obeys adaptation in a Darwinian evolutionary sense, which means that it evolves and adapts: in the case of the brain we should observe a *dynamic neurocognitive adaptation*^5^.

As previously noted, the idea of BEN in neuroscience is related to the idea of uncertainty, which is in turn linked to many other concepts in physics, chemistry and biology, including randomness and information theory (Shannon, 1948), which produces several different definitions (Vaillancourt and Newell, 2002).

The BEN measures the uncertainty of neuronal fluctuations across time, whereas free-energy measures the uncertainty of beliefs encoded by neuronal fluctuations (Carhart-Harris and Friston, 2019). According to the free energy principle (FEP; Friston et al., 2006) the brain is an open, adaptive, complex system far from equilibrium and as with any adaptive self-organizing biological system in nonequilibrium steady-state with the environment, it must reduce its free energy to resist a natural tendency to disorder (Ashby, 1947; Friston, 2010). Self-organized systems move from disorganized to organized structure-function. In this manuscript, through the review of the studies in this field, we point out that even though the brain must reduce its free energy, avoiding disorder, it must also maintain a certain degree of complexity, meaning a dynamic equilibrium in variety and criticality. This complexity will be reviewed both in studies of neuronal fluctuations across time and in research works that have used functional connectivity (FC) exploration during resting state. Although the necessity of the system is to reduce the entropy, the goal does not seem to be the “dark room” (Friston et al., 2012b), or “simplicity,” or negentropy, rather a difficult balance between order and chaos in the specific environment of the agent. Using the words of Merleau-Ponty (1963):

“*Each organism, in the presence of a given milieu, has its optimal conditions of activity and its proper manner of realizing equilibrium”* and (each organism) *“modifies its milieu according to the internal norms of its activity*” (pp. 148, 154).

Since complex cognitive functions are globally organized in the neural system, and arise from elemental functions that are locally organized (Luria, 1980), cortical functions reflect this organization in global and local milieu (Bressler and Kelso, 2016). In fact, according to Edelman (1987), the most fundamental brain operations are integration and segregation. The neural complexity introduced by Tononi et al. (1994), describes anatomical and physiological differences in FC between local segregation and global integration during perception and behavior, where segregation means statistical independence of small subparts of the system (the parts of the system behave independently) and functional integration is the significant deviations from independence, measured through statistical entropy and mutual information (the model behaves as one)^6^.

Shannon entropy’s equation is a method to estimate the average minimum number of bits, or events (in terms of information), needed to encode a string of symbols, based on the frequency of the symbols.

(1) $$H=-\sum_{i=1}^{n}p\,i\times {\text{log}}_{2}\,\left(p\,i\right)$$

Where *H* is the entropy and *p*_*i*_ is the probability of a given event.

The Information Theory (Shannon, 1948) provides a method to measure how much information we have after we receive a message, knowing that with any new message the goal is to know more (have more information) than we did before. The entropy is the average of information (uncertainty) inherent to any message released. The less likely, the greater is the information.

In terms of FC when connections between regions of different networks are poor, those regions are segregated, with no efficiency in their mutual interaction. Conversely, numerous connections between regions of different networks lead to a globally integrated, synchronized network, without segregation within the single parts, as in the case of an epileptic seizure, where the activities of many neurons are highly correlated and strongly coordinated (Beggs and Timme, 2012), but less differentiated and informative. According to the most influential theories in cognitive neuroscience the neural correlates of consciousness can be traced in temporally evolving dynamic processes (Tononi, 2008; Dehaene and Changeux, 2011; Northoff and Huang, 2017; Solms and Friston, 2018). One of these theories by Giulio Tononi (see Tononi, 2012, for an exposition of the theory) claims that every conscious experience must be informative, differentiated and integrated. When brain’s regions become disconnected, as occurs for example during anesthesia, consciousness fades.

Complexity is achieved in systems where integration and segregation are balanced and coexist. A complex system can combine the presence of functionally specialized (segregated) modules with a strong number of intermodular (integrating) links (Rubinov and Sporns, 2010). The human mindbrain system has a larger repertoire of potential neurocognitive states than other species, and this factor is a key property of its greater complex behavior (Tononi, 2012). One of the features related to this greater complexity is an entropy-extension, rather than an entropy-reduction, as one of the processes of human consciousness evolution, subsequently followed by entropy-reduction, through a reorganization of the system (Carhart-Harris et al., 2014).

As previously noted, the idea of entropy originates from thermodynamics and the term *psychodynamics* (thermodynamics applied to the mindbrain system) itself was introduced in 1874 by Wilhelm von Brücke, a known German physiologist and Freud’s supervisor at University of Vienna. Together with Hermann von Helmholtz, Brücke proposed that all living organisms are energy-systems, responding to the same thermodynamics laws. Since Freud was both a Brücke student and a deep admirer of Helmholtz, he adopted his *psychodynamic* view of the mindbrain as a natural extension of the thermodynamic approach. The connection between physics and neural sciences finds one of the most important precedents and founder in van Helmholtz (1962) and his thermodynamic view applied to the brain functioning, as we see further in the next sessions.

Entropy is described by the second law of thermodynamics. The first law is about the conservation of energy among processes, claiming that the quantity of energy in a system remains the same. In other words, energy cannot be created or destroyed in the universe. The second law of thermodynamics describes entropy, concerning the directionality of energy: a direction naturally moving toward a more disordered (entropic) state in any isolated system, meaning that the process will naturally proceed from lower to higher entropy, with a trajectory which describes the arrow of time, as a measurement of entropy within a system. Even if the total amount of entropy in a system is not easy to calculate, it is easier to measure entropy’s variation within it. For a thermodynamic system in which there is a heat transfer of size Q at a temperature T, entropy—represented in this case by S—as two functions of state, can be measured as:

(2) Δ⁢S=Δ⁢Q/T

However, in this case the measurement of changes in the entropy level is relative to systems that are idealized, simple, microscopic, and isolated. These features are abstract, not suitable for biological living systems, which are usually open and complex. In fact, as recalled by Schrödinger (1944) and von Bertalanffy (1969/2009) living systems do not obey the second law of thermodynamics.

Prigogine (1967) pointed out the irreversibility of all natural processes, highlighting that irreversible conditions far from equilibrium (steady-states), may originate spontaneously and may transform from disorder (thermal chaos) into order (*negentropy*), emphasizing the interaction of a system with its surroundings. Unlike thermodynamics, cognitive neuroscience works with complex systems. When entropy increases, the arrow of time progresses toward disorder, but according to Prigogine, at some point it may achieve the appearance of order, despite a loss of potential. He called the structures resulting from an irreversible process *dissipative* to emphasize that they exist only in open systems far from equilibrium, in conjunction with the environment, with fluctuations, and a nonlinear interaction mechanism (Prigogine and Stengers, 1984). As recalled by Buzsáki (2019) using the logic of “*entropy explains time*,” time should move backward in biological systems since in self-organized system entropy decreases (p. 247). Into this theoretical framework comes the FEP, where all biological systems are driven to reduce an information-theoretic—not in thermodynamic sense—quantity known as “free energy” (Friston et al., 2012a), where in open systems we focus on minimizing the related concept of free energy rather than the maximizing entropy. The equilibrium comes between energy and entropy (Prigogine and Stengers, 1984, p. 126). Again, the FEP considers the system in informational rather than thermodynamic terms.

In this manuscript we reflect on studies about BEN, especially in aging, considering the mindbrain system and *neuroaging* itself as a natural, biologic, complex, and irreversible process with nonlinear interactions and a source of oscillations and spatio-temporal organization. As we address in more details in the forthcoming sections, the complexity becomes super-critical in case of primary states. On the other hand complexity naturally decreases during aging and it is abnormally reduced during neurodegeneration, becoming “pathologically sub-critical.”

### BRAIN ENTROPY IN DIFFERENT STATES OF THE SYSTEM

As Mattei (2014) highlights, neuroscientific studies use nonlinear methods to explore patterns of cell firing (Thomas et al., 2013), autonomic systems (Tseng et al., 2013), synchronization of neural networks (Yu et al., 2011), EEG data (Abásolo et al., 2007), and noise modulation in the cerebellum (Tokuda et al., 2010). Neuroimaging approaches can explore function and variability of the brain through different methods. As we noted, it can be calculated in an information theoretic sense, as the magnitude of entropy in a given framework of spontaneous brain activity, such as oscillations in electrical potentials recorded with EEG or MEG. It can also be assessed with the BOLD rs-fMRI signal (Ogawa, 1990) as temporal dynamics of neuronal activity. The BOLD signal explores brain fluctuations self-organized into internally coherent spatiotemporal patterns of activity, an expression of neural systems engaged during different cognitive states (called intrinsic or resting state networks—RSNs; Fox and Raichle, 2005). These networks are spatially segregated brain regions, intrinsically co-activated and deactivated across time. This synchronized activation gives rise to FC as temporal correlations between spatially distinct neurophysiological events (Friston et al., 1993) in which multiple cerebral regions, even those anatomically distant, are activated and deactivated simultaneously during both task and resting states (Biswal et al., 1995). These temporal fluctuations of brain activity are an intrinsic property of the system which underlies brain communication within a specific RSN and between different RSNs. Nonlinear statistical measures are used to calculate the regularity of biological signals, with neuroimaging approaches that use the concept of entropy to measure brain complexity. Although we focus our attention on aging, we first introduce the entropic brain hypothesis through the states in which it has been proposed.

### Brain Entropy in Psychedelic States

The entropic brain hypothesis (Carhart-Harris et al., 2014; Carhart-Harris, 2018) first arose to answer the question: “*what happens to human neurocognitive functionality when non-ordinary states occur?*” The neurodynamics of primary states^7^ are more entropic than secondary states. In fact one of the most important actions of psychedelic compounds is to increase BEN, with a direct behavioral effect of increasing the richness of conscious experience (Carhart-Harris and Friston, 2019). The psychedelic state is considered an archetype of primitive states of consciousness that preceded—from an ontogenetic and phylogenetic view—the development and evolution of modern, human, adult, ordinary waking consciousness (Carhart-Harris et al., 2014). Under the effect of psychedelic drugs such as psilocybin and lysergic acid diethylamide (LSD), the brain reaches greater complexity, compared to normal waking consciousness, giving rise to a more critical activity, as shown by recent fMRI studies (Atasoy et al., 2017; Muthukumaraswamy and Liley, 2018; Varley et al., 2020). These studies support the reduced hierarchy in favor of an increased anarchy activity, with a scale-free organization effect (Carhart-Harris and Friston, 2019). In fact one of the critical effects of psychedelics is a loss of synchronization and a decreasing of oscillatory power in higher level cortical regions via serotonin 2A receptors (5-HT2AR) mediated excitation of deep-layer pyramidal neurons in these regions (Muthukumaraswamy et al., 2013; Tagliazucchi et al., 2014). Psychedelic compounds in fact, upregulate 5-HT2AR and this mechanism can facilitate adaptation to the environment, which is continuously changing and variable (Carhart-Harris and Nutt, 2017). One example is by promoting divergent thinking, favoring psychological flexibility, creating new and effective cognitive, emotional, and behavioral strategies (Kuypers et al., 2016) and impairing conventional cognition (Bayne and Carter, 2018). In this context it is interesting to note that, consistent with our hypothesis, divergent thinking and creativity has been recently correlated with BEN at the level of the left dorsal anterior cingulate cortex/pre-supplementary motor area and left dorsolateral prefrontal cortex (Shi et al., 2020). Divergent thinking seems to be related to the functional dynamics of the control networks involved in cognitive flexibility and inhibitory control.

Analyses of Lempel-Ziv complexity through MEG, measuring spontaneous neural activity effects of LSD and psilocybin and psychedelic-like drugs, have shown increased BEN in psychedelic states (Schartner et al., 2017). Importantly, the magnitude of this higher entropy correlates with the subjective intensity of the drug experience (Schartner et al., 2017). Similar results were obtained using fMRI, again under psychedelic-like drugs, where the magnitude of increased BEN predicted subsequent alteration in personality two weeks later (Lebedev et al., 2016). Further support through fMRI has been observed in studies with ayahuasca (a South American psychotropic plant tea, containing dimethyltryptamine), which showed increased brain complexity under the effect of this compound, using EEG and the Lempel-Ziv measure (Viol et al., 2017; Timmermann et al., 2018).

FMRI studies, focused on psilocybin effects, confirm an expanded repertoire of brain states under LSD and this effect correlates, again, with the experience and the intensity of the subjective “trip” (Atasoy et al., 2017).

The RSNs which exhibited the most significant changes, correspond to higher brain systems such as the Default Mode Network, or Default Network (DN^8^; Raichle and Snyder, 2001), executive control and attention networks rather than primary sensory and motor networks. This outcome is consistent with the regional distribution of 5-HT2A receptors (Tagliazucchi et al., 2014), as we noted, strictly related to the action mechanism of these compounds.

Adult mindbrain in normal and awake conditions works through a “critical-behavior.” In other words the activity of the brain continuously transits between two phases: one in which the brain action increases and amplifies over time and another in which the activity rapidly reduces and dies (Beggs and Plenz, 2003). The transition between these two phases seems to be associated with ordinary consciousness state of “neurocognitive efficiency”: the system has an internal (unconscious) model of the world and through the active inferences, create predictions about expectations. The difference between predicted sensory information (prior probability) and the real sensory information (posterior probability) gives rise to a prediction error (Hohwy, 2013), where surprise, or free energy, can be decomposed into complexity minus accuracy (Cieri and Esposito, 2019).

Studies on the investigation of the LSD effects on brain networks (Preller et al., 2019) using rs-fMRI have confirmed reduced top–down flow from the posterior cingulate cortex (PCC) to the thalamus. In other words, it seems that compared to psychedelic states, in normal conditions subjects are more able to integrate and interpret external information from the environment, and predict errors, using “regular” bottom-up sensory cortices and heteromodal association top-down cortices with their higher value of intermodularity. The thinking style of normal states can have access to the analytical and convergent expression. On the other hand, the psychedelic-primary states move the neurocognitive activity toward a state of increased BEN (i.e., neural activity is more random and cognition is more “anarchic”) without possibility of access to forms of analytical and convergent thinking. Primary consciousness is associated with unconstrained cognition and less ordered (higher-entropy) neurodynamics, whereas secondary consciousness is associated with constrained cognition and more ordered neurodynamics.

Relaxed beliefs under psychedelics (REBUS; Carhart-Harris and Friston, 2019) describes an anarchic neural activity, in which from one hand, there is an increased bottom-up signaling, more sensitive to external (environment) and internal (somatic, visceral, and emotional-limbic) stimuli. On the other hand, the system is exposed to a decreased top-down sensory inhibition, meaning less perceptual restriction. These two dimensions of the system are mutually dependent and critical in their dynamic balance.

There are other human conditions with a negative correlation with BEN, or in other words closer to sub-level of criticality, such as mood disorders (e.g, depression; Akdemir Akar et al., 2015), but the results on this field are contradictory (Mendez et al., 2012) and perhaps suggest that the relationship between BEN and depression maybe subtype and state specific (e.g., see Akdemir Akar et al., 2015), as well as sensitive to medication status (Mendez et al., 2012). The analysis of these conditions is beyond the scope of this work. Nevertheless, there are some analogies that appear evident between psychedelic states and psychotic traits and states—such as schizophrenia. These analogies concern for example a direction of divergent thinking and/or reduced analytical abilities (Kuypers et al., 2016). In the next section we describe the BEN in these psychotic traits and states. Compared to acute psychedelic states, BEN is more problematic to investigate in these conditions because they are more shaped by the interaction of biopsychosocial factors, thus they are as multifaceted as human personality can be. On the other hand, psychedelic states have the power to channel and identify a neurocognitive effect more homogeneously thanks to the acute effect of the compound used, albeit influenced by the biopsychosocial factors. In case of states or traits of psychosis, caution is needed and general and conclusive statements should be avoided.

### Brain Entropy in Schizophrenia

The dysconnection hypothesis (Friston and Frith, 1995; Friston et al., 2016) postulates that the neural correlate of schizophrenia comes from an abnormal interaction between specialized brain regions, resulting in defective integration of activity in distributed networks, and therefore in cognitive disintegration. According to Friston et al. (2016), from a system perspective in psychosis we observe an abnormal balance of synaptic efficacy that mediates the (context-sensitive) influence of intrinsic and extrinsic (long-range) connectivity. Since we need our brain for our active inference, the disconnection hypothesis postulates that the psychopathology must be evident as a failure of the inference process, due to a defect in the modulation of sensory states. It means that the individual cannot ignore stimuli. Statistically speaking everything becomes surprising, because not predictable (Friston et al., 2016). From a synaptic view there is a gain when we have a synchronous exchange of neuronal signals (Chawla et al., 1999). In schizophrenia, synaptic pruning seems to be abnormal from puberty, snipping off far too many dendritic spines (Feinberg, 1990; Glausier and Lewis, 2013; Kandel, 2018).

Bordier et al. (2018), recently used the graph-theory approach to demonstrate that in patients with schizophrenia there is an abnormal connectivity organization in terms of participation coefficient^9^, with higher values in sensorimotor and primary visual cortices compared to healthy controls (HCs). In healthy subjects this value is higher in heteromodal association cortices—with the aim to integrate information—and lower at the level of sensory cortices (Fornito et al., 2015). Conversely, in patients with schizophrenia, more disconnection is found in frontal and parietal cortices, including heteromodal and associative areas, compared to HCs. As in the case of acute psychedelic conditions, the brain’s top-down functions fail in predicting errors in schizophrenia (Carhart-Harris and Friston, 2019).

The vision system might play a key role in this context. In fact, as human being, our surviving and development largely depend on our complex process of sight, which convey information from the environment, on which we base a good part of our movement, orientation, active inferences and behavior. Interestingly, in this regard, Silverstein et al. (2012) and Silverstein and Rosen (2015) have found an anticorrelation between schizophrenia and congenital blindness, recently confirmed by Pollak and Corlett (2019). As shown by these authors congenital blindness seems to act as a protective factor against schizophrenia. The last study used a Bayesian prediction error minimization model, exploring how congenital blindness may increase precision (and consequent stability) of higher-level (including supramodal) priors, focusing on visual loss-induced changes in N-methyl-D-aspartate (NMDA) receptor structure and function as a possible mechanistic substrate.

The sense of sight is fundamental in the construction of the individual internal world model, especially in our species, where visual inputs have primacy in sculpting prior hypothesis about the reality (Pollak and Corlett, 2019). Accordingly with the mentioned evidence and consistently with our speculation, congenital blindness appears to protect against schizophrenia, showing an increased precision of higher-level priors in people blind from birth, providing a less complex system, which acts as a protective factor against the disease. We agree with the authors of this study that propose that the neurocognitive system of the congenital blind patients have more precision of higher-level priors. Therefore, the irregularities that characterize schizophrenia have less impact on congenitally blindness because of the stability of their higher-level supramodal representations (Pollak and Corlett, 2019). Absence (but not subsequent loss) of visual information might make the integration of top-down active inference and bottom-up information “less complex” or “more manageable,” in congenital blindness, especially in a species in which the sense of sight plays such a fundamental role in its evolution, both from an ontogenetic and phylogenetic perspective. Decreasing the intensity, variety and complexity of stimuli, can lead to a protective effect against schizophrenia, in which greater signal complexity results in defective integration in distributed networks and in cognitive disintegration^10^. In congenital blindness, the visual cortex shifts its function to process auditory and tactile stimuli (Poirier et al., 2006), showing an important role of cross-modal plasticity.

At the level of the multimodal integration networks in congenitally blind patients, there is increased interconnectivity across multimodal integration areas and between modal regions and multimodal integration cortices, compared to sighted controls (Ortiz-Terán et al., 2016). As this latest study points out, visual deprivation results in cortical reorganization which seems to make easier the elaboration of non-visual information and aids in overall perception, saliency and environmental characterization.

Schizophrenic patients could show different level (depth) of *disconnection*: between the individual and the external environment—in terms of behavioral symptoms, such as isolation and social withdrawal; between emotion and knowledge; will and action (Frith, 2007); and an innermost *disconnection* within the parts of system itself (neural networks) in terms of FC (Friston and Frith, 1995; Friston et al., 2016).

Pincus (1991) has introduced the Approximate Entropy (ApEn) in order to calculate the regularity and complexity for the analysis of short and noisy brain signal time series, measuring the normal distribution (Richman and Moorman, 2000). The idea is that the time series in which elements are repeated, give rise to more ordered structures, with a lower amount of entropy. Sample Entropy (SampEn) is a variant of Approximate Entropy (ApEn)^11^ to characterize the complexity within dynamic FC, estimated by applying the sliding-window correlation technique. SampEn was introduced by Richman and Moorman (2000) to reduces the bias of ApEn, to count each sequence as matching itself to avoid the occurrence of ln (0) in the calculations. SampEn avoids the problem of vector self-matching, not including it in the analysis. This technique has been recently used (Jia et al., 2017) to show that BEN of the amygdala-cortical connectivity decreases with advancing age, but this effect disappears in patients with schizophrenia. In other words, these authors have shown the general loss of brain complexity (in terms of FC) related to neuroaging, which will be explored in depth in the next sections, but they also added the evidence that schizophrenia is able to “contrast” this age effect. This study provides a demonstration about more entropy associated with schizophrenia with a reversed trend in aging. Even though a “compensation effect” might be too speculative, it is interesting to note that positive symptoms of schizophrenia physiologically improve with aging (Jeste and Maglione, 2013).

A more recent study (Salman et al., 2019) used dynamic FC, supporting the increased subcortical hyperconnectivity, translated in higher entropy in the whole brain and in the functional domain level of patients with schizophrenia, compared to HCs.

Sokunbi et al. (2014) explored the entropy of the brain signal itself, rather than the complexity of FC, finding global and regional differences between patients with schizophrenia and HCs while performing a social exclusion task. These authors have used two different entropy measures: SampEn and Hurst Exponent (HE), where this latter method is used to calculate the brain complexity, computing fractal complexity of the time series. It measures how persistent a fractal process is, or how self-similar (predictable) a signal is within a process^12^. The results of this study show that patients have more entropic fMRI signal, globally and locally, than HCs, according to both measures used. One of the most significant regions found in this study is the ventrolateral prefrontal cortex, which has been reported in fMRI studies to have a key role in responses to social exclusion (Eisenberger et al., 2003), in emotional working memory and the experience of Self (Smith et al., 2018). Patients with schizophrenia have shown higher entropy of the brain signal compared to HCs, also using EEG approach (Raghavendra et al., 2009). Neuroleptic use seems to decrease the abnormally higher EEG disorganization in these patients; this effect might be due to a dopaminergic modulation effect in frontal regions (Takahashi et al., 2010), which is in line with the disconnection theory of schizophrenia by Friston and Frith (1995). Takahashi et al. (2010) highlight how their evidence of increased BEN is an example of the underlying disorganized spiking activity in schizophrenia, consistently with our view.

## Entropy in Aging

Physiologic processes have a complex organization, operating on regulatory models and feedback loops over multiple scales of time and space (Lipsitz and Goldberger, 1992). These processes interact with one another in a nonlinear fashion (Goldberger et al., 2002; Manor and Lipsitz, 2013), having nonlinear dynamics marked by fluctuations over multiple temporospatial scales (Costa et al., 2002). This dynamic complexity typical of physiologic processes decreases with aging in cardiovascular (Costa et al., 2008), respiratory (Peng et al., 2002), central nervous (Yang et al., 2013), and motor control (Costa et al., 2008; Manor and Lipsitz, 2013) systems, and this loss of complexity in aging is directly associated with more difficulty adapting to stress (Lipsitz and Goldberger, 1992). Lipsitz and Goldberger (1992) claimed that fractals and chaos theory are a useful approach to study these changes. Fractals are irregular structures composed by complex patterns, with the feature of self-similarity, where the structure of the smaller-scale mirrors the form of the larger-scale. We can observe examples of fractals in nature, such as Brownian crystal growth and lightning structure, in the human body, such as the blood vessel of the tracheobronchial tree and neural organization (Zueva, 2015). We can also observe evocative fractal ideas in art, as in some Pollock paints, as “One number 31, 1950” (see Taylor et al., 1999 for an interesting discussion about the mentioned Pollock’s paint and fractals).

During development, the complexity and computational power of the mindbrain system increases along with the complexity of the external environment, in “normal” conditions. The neuroaging process is physiologically related to decreased power to receive and elaborate on information from the external environment. This decreasing is in part related to sensory impairment, which negatively affects the sensory–cognitive interface on which we rely, preventing the correct bottom-up process needed for the update of the prior information of the model (*prior probability*) with new information from the environment (*posterior probability*).

The physiological aging process can affect all of the senses, causing gradual losses to the sensory system. Thus, loss of vision, hearing and olfaction are physiologically linked to the impairment of the neurocognitive system. Impairment in visual acuity, contrast sensitivity, dark adaptation, spatial contrast sensitivity, scotopic dysfunction and general visual processing are common even in people without any specific structural vision pathology, such as glaucoma or cataracts (Schaie, 1996). Vision impairment can affect cognitive function (Alzheimer’s Association, 2013) and the use of eyeglasses to correct vision, decreases depressive symptoms (Petersen et al., 2001), often present in aging cognitive impairment and neurodegeneration (Cieri et al., 2017).

On the other hand, hearing loss is the third most common health disorder in older adults (Blackwell et al., 2014) and in elderly it is associated with 30–40% steeper cognitive impairment compared to subjects without hearing decline and with a 24% increased risk of cognitive impairment (Lin et al., 2013). The hearing decreasing in aging is also linked to faster decreases in the volume of brain regions that are important for spoken language processing and other cognitive functions (McEwen, 2000). There are many evidences that vision and hearing decline are associated with greater risk of cognitive impairment (Lupien et al., 2009). In fact, hearing loss has recently been described as a modifiable risk for Alzheimer’s disease (Livingston et al., 2020).

Along with the mentioned sensory impairment, the olfaction declines significantly during aging, in more than 50% of elderly between 65 and 80 years old and between 62 and 80% of those over the age of 80 (Doty et al., 1984). Starting from 60 years, many features of olfaction and the overall smell function start to decline. This specific sensory impairment is also a typical symptom in Parkinson Disease and longitudinal research has shown that olfactory decline is correlated with higher risk in development of the disease (Haehner et al., 2007). Parkinson’s patients report rates of smell impairment in a range between 75 and 95% (Oppo et al., 2020). Olfaction is not exclusively impaired in patients with Parkinson; it is also an early feature of AD and it has been shown to predict progression from mild cognitive impairment (MCI) to AD (Postuma et al., 2015).

From a functional perspective, sensory impairments might have a causal role in cognitive decline, increasing the cognitive load, limiting the neural resources needed for optimal cognitive performance, decreasing the bottom-up sensory perception, therefore preventing the update of the system through posterior probability. Decline of sensory input could also directly affect brain structure and function, for instance it can overload neurocognitive circuitry from resource demands to address poor signal-to-noise ratios. Moreover, sensory loss may lead to depression, social isolation, and lack of physical activity, which could in turn increase cognitive impairment (Whitson et al., 2018).

**FIGURE 1:**
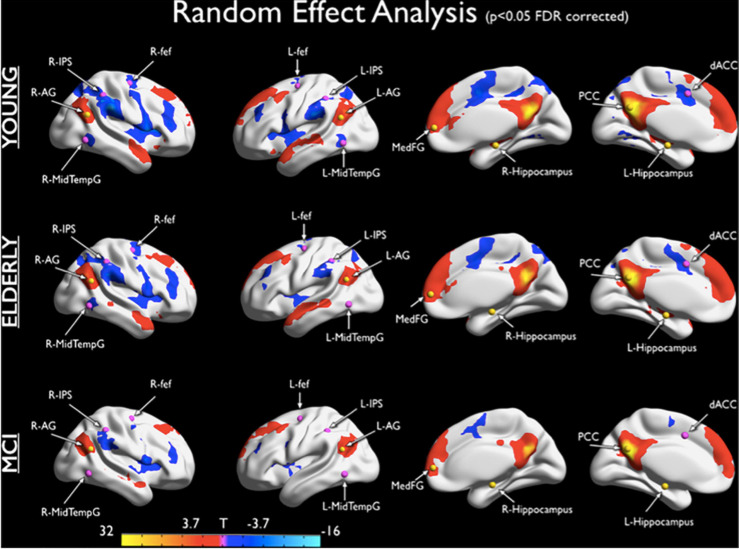
Default Network (DN) and Dorsal Attention Network (DAN) anticorrelations. The maps show DN-DAN anticorrelations for healthy young subjects, healthy elders, and MCI subjects. For each group a positive correlation with the PCC time course is observed in the angular gyrus, Medial Frontal Gyrus and Hippocampus regions, according to topography of DN. For each group, a negative correlation with the PCC time course was observed in the Inferior Parietal Sulcus, Frontal Eye Field, ACC and Middle Temporal Gyrus. These regions overlap with the DAN nodes. This anticorrelation is reduced in elderly and MCI patients compared to young individuals (Esposito, Cieri et al., 2018).

As we see further in the next sections, the evidence shows that with aging we observe an overall decrease of BEN, both in terms of electrical signal complexity and integration-segregation FC of the neural networks, which become less complex. During the neurocognitive aging process (especially in its pathological vein) the system seems to lose its general “*goodness*” to receive information and apply an efficient perceptual synthesis, good factorization of the putative causes of sensations, losing the ability to update, interpret and predict the reality. Associate to this factor there is the progressive loss to anticorrelate specific networks during cognitive task and resting state. In fact, in terms of the fMRI BOLD signal, the young healthy neurocognitive system expresses the mutual inhibition of activity at different cortical regions, leading to anticorrelated dynamics (Dehaene and Changeux, 2011). This ability declines during physiological aging and MCI (Esposito, Cieri et al., 2018; Figure 1). At the same time the system applies some adaptive strategies, such as a more local (segregated) than distributed (integrated) information processing (Heisz et al., 2015).

### Brain Entropy in Physiological Aging

As noted above, the general loss of dynamic complexity associated with aging has been explored in other physiological measures, such as cardiovascular, respiratory, or motor control systems, as well as through different imaging methods such as fMRI, EEG, and MEG. Here we focus our attention to the BEN in physiological and pathological aging, investigated through the rs-fMRI approach (Table 1).

**TABLE 1:** Rs-fMRI studies on BEN in aging.

**Measure**	**Study**	**Stage**	**Groups**	**Results**
Hurst Exponent (HE)	Wink et al., 2008	Young HCs, Elderly HCs	11 young HCs; Age: 22.4 11 elderly HCs; Age: 65.3	Decreased BEN (increased HE) age-related in bilateral-MTL, Hp, amygdala and parahippocampal gyrus
Hurst Exponent (HE)	Dong et al., 2018	Maturation	116 HCs Age range: 19-85	Decreased BEN (increased HE) age-related in frontal and parietal lobes; increased BEN in insula, limbic, and temporal lobe; Sex differences: M > F in parietal lobe
Aproximate Entropy (APEn) + Sample Entropy (SampEn)	Sokunbi et al., 2015	Maturation	86 HCs Age range 19–85 years	Decreased ApEn age-related whole and regional: frontal, limbic, temporal, cerebellum and parietal lobes; No significant correlation between SampEn and age
Multi Scale Entropy (MSE)	Smith et al., 2014	Maturation GM and WM	8 young HCs; Age: 23 8 elderly HCs; Age: 66	MSE increased in GM at higher scales, resembles that of f^–1^ noise, compared to WM; BEN at shortest scale dominated by noise; filtering noise out contrast sharper between GM and WM at longer time scales; more activity in young versus elderly in DN
Multiscale Entropy (MSE)	Yang et al., 2013	Young HCs, Elderly HCs	56 Young HCs; Age: 27.5 99Elderly HCs; Age: 80.6	Decreased MSE in the elderly in the OC, PCC, Hp, SOG, caudate, and thalamus; Most significant in PCC; MSE curve profile shows a decreased MSE with increased scale factors
Shannon Entropy (SE)	Lou et al., 2019	Elderly HCs	188 Elderly HCs; Age: 70.8	Decreased SE age-related in the r-IFG, l-IPG, r-amygdala, r-Hp, left parahippocampal cortex; High BEN values mainly distributed in the frontal and temporal lobes; occipital and parietal regions exhibit a more stable pattern with low diversity values
Approximate Entropy (APEn)	Liu et al., 2013	Maturation and fAD	8 young HCs; Age: 23 8 elderly HCs; Age: 66 22 fAD (PSEN1, APP) Age: 41.2	Decreased ApEn age-related in elderly HCs in bilateral angular gyri, r-MTG, r-SMG and PCC, compared to young HCs; Decreased ApEn AD-related in Prc, right SMG AG, bilateral superior parietal regions; Main effect of age on mean ApEn values in GM more than WM
Multi Scale Entropy (MSE)	Smith et al., 2015	Elderly HCs, MCI, GM and WM	25 elderly HCs; Age: 70 25 MCI; Age: 70	Lower BEN across all scales (dominance of external noise) in WM; High BEN at lower scales, where the noise dominates the entropy and GM not distinguishable from WM; DN highest level of irregularity
Multiscale Entropy (MSE)	Grieder et al., 2018	Elderly HCs, AD	14 elderly HCs; Age: 67 15 AD; Age: 67	Decreased MSE in AD compared to HC increasing scale; DMN-related signal fluctuation less complex in AD at lower frequencies. More entropy in the HC from scales 1–4, followed by a decrease from scales 5–10
Multiscale Entropy (MSE)	Niu et al., 2018	Elderly HCs, EMCI, LMCI, AD	30 elderly HCs; Age: 74.1 33 EMCI; Age: 72 32 LMCI; Age: 72.5 29 AD; Age: 72.3	Decreased MSE in MCI and AD patients associated with cognitive decline on multiple time scales (from 2 to 6) especially occipital, frontal, temporal, limbic, and parietal lobes, compared to HCs; Shortest scale, entropy dominated by high frequency fluctuations from random noise; Filtering out random fluctuations, bigger contrast at longer time scales
Permutation Entropy (PE)	Wang et al., 2017	Elderly HCs, EMCI, LMCI, AD	30 Elderly HCs; Age: 74.1 33 EMCI; Age: 72 32 LMCI; Age: 72.5 29 AD; Age: 72.3	Decreased PE in AD than MCI and HCs especially in the occipital, frontal, and temporal lobes; Significant negative correlations between PE and ReHo in patients at the inferior and middle frontal gyrus; MMSE, FAQ and CDR scores, revealed an increasing symptom load with decreasing PE
Sample Entropy (SampEn)	Wang, 2020	Elderly HCs SMC, EMCI, LMCI, AD	54 elderly HCs; Age: 75.3 27 SMC; Age 72.4 58 EMCI; Age: 71.5 38 LMCI; Age: 71.8 34 AD; Age: 72.4	SampEn slightly increased from HCs to SMC and EMCI, it quickly fell below the BEN level of HCs in LMCI, showing a further steep in AD with an accelerated pace, specifically at the level of the DN and the executive control network (including the dorsolateral prefrontal cortex and lateral parietal cortex)

*BEN, brain entropy; Development, from infancy to adulthood; Maturation, from adulthood to elderly; Age, mean age age; HCs, healthy controls; SMC, significant memory concern; fAD, familiar Alzheimer; SZ, schizophrenic; M, Male; F, Female; PSEN1, Presenilin-1 gene; APP, Amyloid Precursor Protein mutation gene; ReHo, regional homogeneity; GM, gray matter; WM, white matter; Hp, hippocampus; MTG, mid temporal gyrus; SMG, supramarginal gyrus; AG, angular gyrus; PCC, posterior cingulate cortex; Prc, Precuneus; OC, olfactory cortex; SOG, superior occipital gyrus; LG, lingual gyrus; STC, superior temporal cortex; ACC, anterior cingular cortex; IFG, inferior frontal gyrus; IPG, inferior parietal gyrus; MTL, medial temporal lobes; DN, default network; MMSE, Mini Mental State Examination; FAQ, Functional Activities Questionnaire; CDR, Clinical Dementia Rating.*

During aging we observe a general cognitive change in all the cognitive spectrum, including attention, language, memory, inhibitory control and other executive functions. The discussion of these changes is beyond the scope of this manuscript (see Cabeza et al., 2018; but also Grady, 2012, for a review) and we focus our attention to the BEN changes during physiological and pathological aging.

Brain entropy is investigated through nonlinear methods, such as the aforementioned HE, used to estimate the fractal complexity comparing two groups of young and elderly individuals, showing in the latter group decreased entropy of the signal at the level of bilateral hippocampus, as one of the most important and affected regions in aging (Wink et al., 2008). The same approach has been more recently used to calculate the complexity of rs-fMRI during maturation in 116 HCs between 19 and 85 years old, showing a significant association between the mean HE of whole brain gray matter and the age of participants, where decreasing BOLD signal complexity (higher HE) is correlated to aging (Dong et al., 2018). This reduced complexity is particularly focused at the level of the frontal and parietal lobes, but at the same time this study points out an increased complexity at the level of limbic regions, such as insula. The authors have speculated that this increase may be related to the higher involvement of limbic areas in emotional states, which do not become impaired with age and sometimes improve in some aspects (see Nashiro et al., 2012 for the emotion paradox in neuroaging).

Combining SampEn with Fuzzy Approximate Entropy, Sokunbi et al. (2015) explored the brain aging dynamics in maturation in 86 HCs with age range 19–85 years, demonstrating that the mean whole and regional BEN show significant negative correlation with age; specifically, at the local level within the frontal, parietal, limbic, temporal, and cerebellum parietal lobes.

Multi Scale Entropy (MSE; Costa et al., 2002) applies SampEn to different coarse-sampled scales from the original time series, in order to differentiate complex processes from random fluctuations by exploiting differences between signal and noise across multiple time scales, because the noise signal can show a high level of complexity as well. BEN may not be directly interpreted as the degree of complexity, because random noise defers the highest entropy but it does not represent the most complex process (Smith et al., 2014). It is critical to estimate whether the information transferred is the same across scales. MSE differentiate random noise from complex signals, where random noise decreases with longer time scales. This method consists of a set of SampEn values under multiple time scales, which reflects the complexity of time series on multiple scales. Thus, it can be used to compare the complexity of different time series, based on the specific trend of SampEn changes with scales (McIntosh, 2019). MSE has been also used to compare white versus gray matter in young versus elderly by Smith et al. (2014), showing higher BEN in white compared to gray matter at shorter time scales and the opposite at lower temporal frequencies. Both groups young and elderly show complexity at increasing scale. Values of entropy are similar at low scales between young and elderly, with dominance of noise at higher frequencies. Filtering out random fluctuations (noise), the entropy difference between groups becomes more evident in the range of low frequencies, in regions including the DN (Smith et al., 2014).

Older subjects also exhibit significant MSE reduction at the level of olfactory cortex, superior occipital gyrus, and thalamus, compared to the younger group, during resting state (Yang et al., 2013). Importantly, this latter study has shown that the most significant values of reduced entropy are at the level of the PCC, caudate and hippocampal cortex, as important components of the DN, compared to young individuals (Yang et al., 2013). The DN is involved in cognitive functions, such as autobiographical, episodic memory and self-referential processes (Buckner and Carroll, 2007; Cieri and Esposito, 2018). The PCC is part of the posterior nodes of the DN and it shows significant modification in aging, especially pathological (e.g., AD), indicating amyloid deposition and reduction of metabolism, also in preclinical conditions and APOE-ε4 carriers (Sperling et al., 2009; Caldwell et al., 2019). Moreover, the PCC, precuneus, and associated paramedian thalamic nuclei, are suggested as a strategic intersection for coordinating the interactions among different sensory areas and frames of reference concerning the body and the environment. In fact, bilateral lesions to these areas are related to a virtual breakdown of information integration in the thalamocortical system (Tononi, 2004).

Interestingly, McIntosh et al. (2013), using different imaging techniques, such as both EEG and MEG, have explored age differences through MSE and FC, showing that at coarse scales a decreasing of complexity is observed, while at fine scales the opposite occurs. Healthy elderly subjects seem more prone to process information in local rather than distributed ways. The anticorrelation between fine and coarse timescales in the older group suggests a shift in the direction of adaptive local processing in healthy aging. Importantly their cognitive performance, measured by MoCA, seem to be correlated with their physical activity, whereas this correlation is not true for younger individuals (Heisz et al., 2015).

Shannon entropy (SE) has been used to correlate BEN and aging, showing a decrease of age-related complexity in the whole brain as well as in the right inferior frontal gyrus, right amygdala, right hippocampus, and left parahippocampal gyrus at the frequency interval of 0.06–0.12 Hz (Lou et al., 2019). However, this study points out that whole-brain resting state entropy reflects general cognitive flexibility and information processing, measured by typical executive functions neuropsychological tests. The most complex regions found by these authors, were located at the level of frontal and temporal lobes, while a more stable pattern was found in the occipital and parietal regions. The areas correlated with low age-related complexity are mainly located in the DN and frontoparietal control network. Importantly, unlike the other studies analyzed, here complexity is more closely related to a specific cognitive aspect such as mental flexibility, adding a putative neurophysiological mechanism of cognitive flexibility, or at least a closer link between BEN and cognition (Lou et al., 2019). This link is consistent with the general decrease of the neurocognitive flexibility in aging found both on a cognitive level and on a neural signal level.

### Brain Entropy in Pathological Aging

If the decrease in entropy is associated to a general aging of the system, which loses its optimal functionality, then we should be able to observe even a greater loss of entropy in a system affected by neurodegeneration, such as AD (Table 1). Beyond schizophrenia, AD represents another example of a disconnection syndrome (Geschwind, 1965; Delbeuck et al., 2003; Contreras et al., 2019). Participation coefficient and degree centrality^13^, as measures of connectivity of the graph theory approach, have shown that brain regional nodes with the highest number of connections (edges) to other regions are the most frequently lesioned by several brain diseases, including schizophrenia and AD (Crossley et al., 2014). The brain regions most critical to intermodular communication are also more likely to be pathologically lesioned. In fact, in AD cortical hubs have been suggested to be critical areas, concentrating most of the amyloid-β deposition (Buckner et al., 2009). Moreover, in the transneuronal tau propagation hypothesis in AD, the tau pathology seems to spread through neuronal connections, rather than anatomical proximity (Kaufman et al., 2016; Franzmeier et al., 2020), supporting the essential role of *connection-disconnection* in AD. In these patients, the DN, the salience network and the frontoparietal control network display a significant network isolation, or greater connectivity distances, from the rest of the brain, giving more evidence to the disconnection of the system (Costumero et al., 2020).

A trend of less entropy of brain oscillations in the DN regions has been found using ApEn to explore the BOLD signal complexity of cognitive decline associated with familial AD in gray and white matter compared to HCs (Liu et al., 2013), consistently with studies showing decreasing FC in these areas with aging (Damoiseaux et al., 2006). This FC is further reduced in AD patients and persons at risk for dementia, such as APOE-ε4 carriers (Caldwell et al., 2019).

Some studies have shown a correlation between complexity at low frequencies in specific nodes and FC in the same node (Wang et al., 2017), which is consistent with the idea that the complexity of the signal at low frequencies should be correlated with information transfer between nodes of a network. In contrast, other studies have shown a negative correlation between complexity at low frequencies and FC, suggesting that a less complex signal is more regular and that regularity should increase the probability of a phase relationship between different brain areas, and thereby synchrony and information exchange across distributed regions (Ghanbari et al., 2015). As we noticed the association between entropy and FC is still unclear and much less is the role of the APOE-ε4 genotype in this relationship.

Smith et al. (2015) have extended their initial investigation (2014) of the regularity with which the brain reconstructs temporal activity patterns by measuring the stability of recurring subsequences in the rs-fMRI signal, to subjects with MCI showing similar reduction of MSE, again at the level of regions of the DN, such as precuneus, PCC, angular cortex, and medial prefrontal cortex, of cognitively impaired patients.

Lower levels of entropy in DN regions have been found in most scales (Grieder et al., 2018) in AD subjects, showing a constant reduction of complexity with increasing scales in this form of dementia, compared to HCs and a mean-MSE group comparison that showed decreased right-hippocampus entropy in the AD patients. The latter study has shown distinct patterns between the two groups, with DN-related signal fluctuation less complex in AD at lower frequencies. Specifically, there is more entropy in HCs from scales 1–4, followed by a decrease from scales 5–10. As the authors point out, this distinct behavior between HCs and AD might reflect a disturbed regional functional integrity in the patients group, as it has been proposed that higher frequencies are related to intra-regional processing, whereas lower frequencies are thought to be closely associated with inter-regional FC (McIntosh et al., 2013; McDonough and Nashiro, 2014; Wang et al., 2017; Grieder et al., 2018).

Comparison between HCs, MCI, and AD patients, exploring the BOLD signal differences has shown significant differences, especially at the level of occipital, frontal, temporal, limbic, and parietal lobes (Niu et al., 2018), using the MSE. Specifically, lower BEN found in MCI and AD patients associated with cognitive decline on multiple time scales (from 2 to 6) compared to the elderly healthy group. At the shortest scale, the entropy is dominated by high frequency fluctuations from random noise; filtering out the random fluctuations, the bigger BEN contrast is at longer time scales. HCs have shown higher entropy than MCI, and MCI had higher values than AD patients, supporting the hypothesis that BEN decreases with age, especially in cognitive pathological aging, such as MCI and AD.

Decreased complexity in AD patients has been also found again at the level of the medial prefrontal cortex of the DN regions (Wang et al., 2017), using SE applied to permutation vectors (permutation entropy; Bandt and Pompe, 2002). This technique analyzes the irregularity of non-stationary time series considering only the ranks of the samples, not their metrics. Again, the last work has shown decreased complexity in MCI and AD patients compared to elderly cognitively HCs, with an interesting and significant increase of the symptom load with decreased complexity of the brain (Wang et al., 2017).

Another recent paper (Wang, 2020) confirmed that the BEN decreased in the AD continuum. The authors have used SampEn, showing that although the BEN level slightly increased from cognitively healthy aging to significant memory concern and EMCI, it quickly fell below the BEN level of HCs in LMCI, showing a further steep in AD with an accelerated pace, specifically at the level of the DN and the executive control network (including the dorsolateral prefrontal cortex and lateral parietal cortex).

The evidences seem to support a trajectory in which there is a loss of complexity during aging, especially in the cognitive pathological form, such as MCI and AD. In the next section, we interpret these results in the light of the FEP and the idea of criticality.

## Ben Criticality and the Free Energy Principle

According to the FEP the mindbrain system tries to reduce entropy, but consistently with the studies reviewed, the aim of the system it is not the “dark room,” *simplicity*, as the opposite of complexity, or negentropy as the opposite of entropy. In fact, neurocognitive systems that predict complex environments will find the “dark room” surprising and will leave at the earliest opportunity (Friston et al., 2012b). In a healthy and awake condition, the adult individual mindbrain system must be able to maintain a dynamic and complex level of *criticality*, where the idea of criticality is intended as a transition “zone” rather than a static and fixed point (Moretti and Munoz, 2013; Carhart-Harris et al., 2014).

The fact that the brain is considered a system that wanders near a critical dynamic zone between states of order and disorder is widely accepted in cognitive neuroscience (Chialvo, 2010; Carhart-Harris et al., 2014; Tagliazucchi, 2017). In fact, the system self-organizes under normal conditions into transiently stable spatiotemporal configurations (Mantini et al., 2007; Deco and Corbetta, 2011) and this instability is maximal at a point where the global system is critically poised in a transition zone between order and chaos (Tononi et al., 1994). In the current context, the “metastability” (Tognoli and Kelso, 2014) of a neural network is a measure of the variance in the network’s intrinsic synchrony over time.

The survival of a species depends on its adaptive capability to the environment, which in turn depends on the ability of members to react adequately when the environment changes. Although the neurocognitive system needs to resist disorder, it does not mean that it works better without complexity. Members (and groups) that express more variability have more probability to survive than members (and groups) with less variability. In this framework, neurodynamics of modern healthy adults, work better at a critical level, whereas primary states are more “entropic,” meaning that they work at super-critical levels, exhibiting more pronounced characteristics of surprise. On the other hand, with aging we observe a natural reduction of this criticality, which becomes extremely sub-critical in pathological aging such as AD.

Free energy principle in the neurocognitive system is linked to BEN in an information theoretical sense, where the brain tries to resist disorder, making assumptions and interpretations on reality, through active inference (Friston et al., 2012a). This makes the human neurocognitive model a hypothesis testing system (Gregory, 1980), in which it tries to guess the best statistical approximation (generative models) of the causes from the sensorial apparatus, through the Bayesian approach, updating the system (Friston, 2010). van Helmholtz (1962) claimed that perception of the world is not direct, but instead depends on unconscious inferences, or expectations that model perceptions, even before we can consciously perceive any specific object. He called the “*likelihood principle*” a predictive coding approach, also eloquently described by Chris Frith (2007) as “*a fantasy that coincides with reality*.”

Free energy principle comes from the Helmholtz view and the computational work of Dayan et al. (1995), where the brain tries to make sense of the world, anticipating reality through predictions, active inferences, representations of the environment based on personal experience and expectations, learning from experience, with the goal of limiting the entropy, the uncertainty. Any self-organizing system, including the human neurocognitive model, resists the distributed effects of a natural increase in entropy for its existence, development, and evolution, by trying to minimize free energy (Friston, 2010; Cieri and Esposito, 2019). Our system has evolved with the imperative to decrease the uncertainty within the system and between the system and the outside, with instructions for neuronal messages to interpret the reality and update constantly its own model. The brain has a model of the world and tries to update it using new information from sensory inputs (van Helmholtz, 1962; Gregory, 1980; Ballard et al., 1983; Friston et al., 2006). The mindbrain system works as a Helmholtz, or inference machine, performing inferences according to the Bayesian approach, updating the prior information of the model with new information from the environment. Here the Bayesian estimation success in matching will increase the reliability of the model (increasing the posterior probability), while failure will decrease it with an increase of free energy and uncertainty. The automatic activity of the system in terms of prediction or active inference gives rise to a discrepancy between perceptual data from the external world and internal representations. This discrepancy, which is the divergence between complexity minus accuracy, produced by Bayesian belief updating is the free energy (Hopkins, 2016). This free energy generates uncertainty or surprise. The aim of the system is adaptation to the complexity generated by reality.

As we have seen, individuals with congenitally blindness show increased stability of higher-level priors, possibly via increased NMDAR-mediated signaling (Pollak and Corlett, 2019), because sense of sight confers consistency (Nardini et al., 2008), especially in a species like us, in which the sight conveys a lot of different information about the external states (environment). In sensory healthy systems visual loss will decline the precision of visual input. In aging, especially cognitively pathological, the sensory system loss its efficiency and the requested increased top-down modulatory signaling—as a way to guarantee stability of higher level priors—is impaired, in a double feedback vicious circle. Given the premise of the mentioned characteristics it makes sense the cited important features of neural complexity as the ability (and the necessity) of the brain to explore alternative states (Friston et al., 2012a). This dynamic criticality (Chialvo et al., 2007), gives the system the opportunity to be more flexible and adaptable to change. This can be seen as a paradox of the system, in which it needs “instability” to be more dynamically and “adaptively stable.”

Complex behavior of neurons enables them to switch readily between different states (Schiff et al., 1994), providing the flexibility needed. This flexibility, at a different scale level, is the key of the neurocognitive system, making it suitable and adaptable. This “self-organized criticality” is seen in complex adaptive systems, as neural networks, able to show a *complex dynamic balance*, in which the system needs to integrate and segregate information. The image emerging illustrates a system that needs a critical world to survive, displaying critical behavior (Beggs and Plenz, 2003; Chialvo et al., 2007). As we noted, critical behavior means that activity of the brain continuously transits between two phases, one in which activity increases and amplifies over time and another in which activity rapidly reduces and dies (Beggs and Plenz, 2003). Between these two phases, there is a critical zone in which the system increases information processing.

In ontogenetic sense, from birth to adulthood, the direction in structural network modules seems to proceed toward a more segregated system, with weaker connections between modules and stronger connections within modules, associated to cognitive development and specifically with an improvement of executive functions (Baum et al., 2017). This is consistent with the cybernetic perspective by Bateson (1972) in which the system is regulated by a constraint that is an economy of alternatives, which is in turn a neural networks application of the Occam razor’s principle, where the system tries to find the easiest possible explanation that provides an accurate account of the sensorium (Tononi, 2012; Friston et al., 2015; Cieri and Esposito, 2019). This more segregated system does not mean isolation. In fact, the healthy young system needs a balance between segregation and integration in terms of FC. For instance, better performances in spatial working memory task seem to be associate with higher global efficiency and modularity, in which the networks show strong segregation, with better integration (short pathways) between its nodes to increase the efficiency in global communication between brain modules (Alavash et al., 2015). The complexity of the system, between segregation and integration, makes flexibility possible; according to this flexibility, RSNs must have the capability to correlate/anticorrelate among them (Esposito, Cieri et al., 2018).

During resting state, the brain cortex prominently expresses α brainwaves (e.g., ∼10 Hz), associated with different functions, including top-down inhibition (Klimesch et al., 2007), top-down prediction and information processing (Mayer et al., 2016). This activity seems to be associated with the DN activity (Mantini et al., 2007), as part of neural correlates of the “ego integrity” (Carhart-Harris and Friston, 2010; Cieri and Esposito, 2019).

One peculiar feature of the system is its dynamic characteristic to wander, or not establish into any specific state with the propensity to destroy its own fixed points—a characteristic named *autovitiation* (Friston et al., 2012a) to emphasize the fundamental role that self-induced instabilities play in maintaining peripatetic, or wandering, itinerant dynamics. Those dynamics in turn enable the aforementioned neural flexibility for correlation and anticorrelation of networks. Since the external environment is complex and changes constantly across time, this complexity affects the neurocognitive system, requiring variability of states for an effective and rapid adaptation to the uncertainty of reality. For this reason, the brain self-organizes its behavior through transiently stable spatiotemporal configurations. This fluctuation reaches its peak when the system is in critical position of transition between order and chaos (Tononi et al., 1994). As we have seen, in primary states this position becomes super-critical during non-ordinary states such as state and traits of psychosis (e.g., schizophrenia) and acute psychedelic state, in which the activity of the system is more entropic compared to secondary states. Both acute and chronic conditions reviewed show a more anarchic brain functioning, with increased bottom-up signaling and reduced top-down sensory inhibition. On the other hand we observe a decreasing of BEN during cognitive physiological and especially pathological aging (e.g., MCI and AD), in which we observe a decreased bottom-up signaling and impaired top-down mechanisms. This decreasing of BEN is consistent with the loss of complexity in cardiovascular (Costa et al., 2008), respiratory (Peng et al., 2002), central nervous (Yang et al., 2013), and motor control (Costa et al., 2007; Manor and Lipsitz, 2013) systems, associated with more difficulty adapting to stress.

The complexity of neural networks seems to grow with the complexity of cognitive and behavioral functions, in a nonlinear dynamic way, where extreme network dynamics might be incapable of sustaining a coherent cognitive flow, showing an “inverted-U” relationship (McIntosh, 2019). The studies taken into consideration on this matter seem to describe a trajectory in which the complexity of the neurocognitive system seems to increase until there is a complexity decreasing in the system (internal and external states) during the neuroaging process, with many age-related changes associated with a reduced dynamic complexity and more difficulty adapting to stress (Lipsitz and Goldberger, 1992). In this trajectory the complexity increases in development and declines with aging, especially in pathological manifestations, such as MCI and AD. Consistently with this hypothesis, the complexity of cognition seems to be maximal around 25 years (Gauvrit et al., 2017), which is in turn consistent with the peak presence of α brainwaves (Basar and Güntekin, 2009), and the serotonin 2A receptor densities (Sheline et al., 2002).

## Discussion

“*The brain is an information-processing organ made marvelously powerful not by its mystery but its complexity* (Kandel, 2006; p. 6).

Entropy is the difficulty in predicting one or more results, inversely proportional to the power to predict a pattern, a given state, or a signal within a specific state. It measures the randomness and predictability of stochastic processes. In this manuscript we argued that during ontogenetic development, complexity of the environment and consequently of the brain, naturally increase in order to adapt to the changing environment. In primary states the BEN increases over level of criticality, and during cognitive aging this complexity decreases.

Even evidence that showed decreased BOLD signal complexity in regions such as frontal and parietal lobes, have found, on the other hand increased complexity in other regions (Dong et al., 2018) such as insula, limbic, and temporal lobe in elderly individuals, consistent with the idea of age related changes in regions more typically involved in more “cognitive” task, but not in regions more typically involved in more “emotional” reactions, showing a possible association to the *emotion paradox* in neuroaging (Nashiro et al., 2012).

The greater repertoire of potential mental states of the human neurocognitive system comes from the complexity of the environment and the ability to interact with it. This relationship between systems affects the complexity of the brain, which operates in turn on the complexity of the environment in a double feedback mechanism. Evolution, as well as individual learning, can act in a similar manner on the brain, because the evolution can also be formulated as learning statistical structure in the environment and distilling that structure into the phenotype (Paulin, 2005; Friston and Buzsáki, 2016). In this context, the ontogenetic increase of BEN during development seems to *recapitulate* the phylogenetic increase. Humans have the most complex cognition and behavior, compared to other animal species. Similarly, humans have the most prominent presence of α brainwaves, compared to other mammals and primates (Basar and Güntekin, 2009) and the adult healthy brain have the higher neural complexity and cognition (Gauvrit et al., 2017). Studies of comparative neurosciences have shown that two human frontoparietal networks may be evolutionarily novel (Dehaene and Changeux, 2011; Mantini et al., 2013), where phylogenetic changes in terms of expansion and complexity may underlie the acquisition of novel cognitive abilities during evolution (Duncan et al., 2000; Dehaene et al., 2003). Particularly important for our context, it has been proposed that the DN has expanded its functions in support of spontaneous cognition (Mantini et al., 2011). An entropy-extension as one of the processes of human consciousness evolution, subsequently followed by an entropy-reduction (Carhart-Harris et al., 2014) might be a specific characteristic adaptable both in phylogenetic and ontogenetic sense.

Buzsáki (2019), claims that “the mystery is always in the middle,” speculating that the diversity of the brain, in terms of neuronal firing rates, connection strength between neurons, and the magnitude of their concerted action—which can vary by three to four orders of magnitude and respects a logarithmic rule—is the essential backbone that provides stability, resilience, and robustness to brain networks (p. 25). Criticality is known to confer functional advantages to a system in terms of maximizing the capacity and efficiency of information processing through optimizing adaptability while preserving order (Shew and Plenz, 2013) and it stands to reason that a system moving closer to criticality and/or shifting closer to the super-critical end of a critical zone is likely to favor flexibility and susceptibility to perturbation over preservation as well as exploration over exploitation (Cohen et al., 2007).

The word *entropy* is composed by two Greek words “*en*,” which means inside and “*trope*,” meaning transformation^14^. Although it might be a mistake try to solve a topic whose complexity is ingrained, in a simplistic way, it seems reasonable to expect a general increasing of complexity during individual development, when we observe a continue *transformation* of the external and the internal states, associated with a general increase of stimuli from internal and external environments. Likewise, the aging process—especially in its cognitively pathological vein—seems to be associated with less complexity, related to a reduction in the sensory ability to receipt stimuli in a bottom-up way and cognitive capability to process interpret and integrate those stimuli in a top-down manner.

## Limitations and Future Directions

The first limitation of the investigation of BEN through rs-fMRI is that this topic is still in its infancy, especially with the FEP approach. Furthermore, these data contains a large fraction of noise, from multiple sources such as head-motion and physiological oscillations. Also, rs-fMRI article reviewed in the current manuscript focused on the FC (both static and dynamic) and the related BEN changes, which do not involve any directionality in the connectivity. A possible future direction might be focusing in studies of effective connectivity and the related BEN changes, more adapt to explore the neuronal basis of metastability, through the dynamic causal modeling (Zarghami and Friston, 2020).

Moreover, although, as we have seen most evidence speaks in favor of a general loss of complexity with aging, there are some exceptions (Vaillancourt and Newell, 2002; Yao et al., 2013) which have proposed that complexity of physiologic signals might increase in aging. Among others, the great presence of noise, the specific method used, global versus regional results, age range and time scale, exist for these discrepancies. Changes in complexity cannot be characterized in a simplistic and unidirectional way, depending on the spatiotemporal dynamics (McIntosh, 2019).

Future directions in this field might include the exploration of the BEN and complexity with development, aging and psychopathology, through different time scales (coarse and fine). In addition, the investigation of BEN and its association with FC may yield interesting results.

Another important future direction might be the study of BEN in its association with genetics. We know that genetics plays a role in AD, for example in the case of the APOE, where the ε4 carriers can be associated with amyloid deposition and reduced FC at the level of DN. We also know that in schizophrenia we can have genetic components in schizophrenia, such as the pathogenetic implications for NMDA receptors and their interactions with neuromodulatory transmitters. In the next future it will be important take in consideration these features correlated to the BEN.

## Data Availability Statement

The original contributions presented in the study are included in the article/supplementary material, further inquiries can be directed to the corresponding author/s.

## Author Contributions

FC Study conception and design and draft manuscript preparation. XZ revised the manuscript critically for important intellectual content. JC revised the manuscript critically for important intellectual content. DC revised the manuscript critically for important intellectual content. All authors reviewed the results and approved the final version of the manuscript.

## Conflict of Interest

The authors declare that the research was conducted in the absence of any commercial or financial relationships that could be construed as a potential conflict of interest.

## Footnotes

1 In this manuscript we use the term *neurocognitive* or *mindbrain* system interchangeably, meaning that the brain (neuro) and mind (cognitive) are two aspects of the same “dimension” according to the *dual aspect monism* (Solms, 1997; Solms and Turnbull, 2003; Atmanspacher and Fuchs, 2014; Solms and Friston, 2018; Solms, 2019).

2 Although the Markov blanket idea was born as a statistical tool used in machine learning, performing a useful function in artificial Bayesian networks, in this manuscript we adopt and endorse the Fristonian concept (expressed for example in Allen and Friston, 2013; Clark, 2017; Friston, 2018; Kirchhoff et al., 2018; Cieri and Esposito, 2019; Ramstead et al., 2019), in which the idea of Markov blanket is extended further and wider to almost every level of organization in nature, from cells to brains and beyond to populations and even entire societies and cultures. From a biological perspective, for instance the plasmalemma is the natural Markov blanket of the neuron, able to make possible both the differentiation and the communication between the internal and external states (Cieri and Esposito, 2019; Ramstead et al., 2019). Currently there is an active and important discussion about whether this construct should be interpreted in a realistic way, applied to biological systems (Bruineberg et al., 2020; van Es and Hipólito, 2020; Beni, 2021).

3 For example, with reentrant interactions among widely distributed brain regions (Edelman, 1993).

4 We can summarize this characteristic with the gestaltic motto: “*the whole is more than the sum of its parts.*”

5 For an interesting discussion on the dissipative structures in biological systems see Goldbeter (2017, 2018), in which he discusses biological rhythms as specific source of dissipative structures in biology.

6 Entropy and mutual information are considered only in their statistical meaning (as the multivariate generalizations of variance and covariance in univariate statistics; Tononi et al., 1998).

7 We are referring to the processes described by the Freudian metapsychology (Freud, 1940), in which Freud distinguished two ways of mental functioning: the primary and the secondary processes, corresponding to the pleasure and reality principle, respectively. Friston (2010) calls it secondary consciousness, which pays deference to reality by carefully sampling the world and learning from its encounters (Carhart-Harris and Friston, 2010; Cieri and Esposito, 2019).

8 According to Andrews-Hanna et al. (2014) we use *default network* rather than *default mode network*, because the *mode* part refers to a more passive state. We want to point out the adaptive functions of the network (extend beyond the resting state), in ontologic and phylogenetic sense, especially important in our context.

9 A method that measures the diversity of a single node, or its intermodularity. Nodes with high values represent more projections to other modules.

10 This hypothesis can be conceivable only in case of congenital blindness; otherwise for late onset of blindness the possibility of developing hallucinatory episodes, is greater, which is in turn consistent with our speculation, because in this case a new unexpected uncertainty of the system comes suddenly, thus the surprise increases in a system not “prepared” for the new disordered condition.

11 Both ApEn and SampEn have the aim to estimate predictability within a time series. ApEn has the characteristic of dependence on data length, while SampEn is data length independent.

12 The value of HE is expressed in a range of time series between 0 and 1, where 1 = 0.5 represents a random white noise series; 2 = 0 < H < 0.5 is a rough anticorrelated series, and 3 = 0.5 < H < 1 describes a positively correlated series (Tagliazucchi et al., 2013; Sokunbi et al., 2014).

13 Degree centrality is a measure of centrality, which calculates the number of connections (i.e., edges) of a node, distinguishing hubs and non-hubs.

14 The word was introduced by Rudolf Clausius, who decided to use the letter *S* to indicate *entropy* in honor of Sadi Carnot, a theoretical father of thermodynamics.
